# Recent Developments in Optofluidic Lens Technology

**DOI:** 10.3390/mi7060102

**Published:** 2016-06-10

**Authors:** Kartikeya Mishra, Dirk van den Ende, Frieder Mugele

**Affiliations:** Physics of Complex Fluids Group, MESA+ Institute, University of Twente, P.O. Box 217, 7500 AE Enschede, The Netherlands; k.mishra@utwente.nl (K.M.); h.t.m.vandenende@utwente.nl (D.v.d.E.)

**Keywords:** optofluidics, liquid lenses, photonics, optical MEMS, micro-optics, adaptive optics, optical aberrations, spherical aberration, lens characterization, wavefront sensing

## Abstract

Optofluidics is a rapidly growing versatile branch of adaptive optics including a wide variety of applications such as tunable beam shaping tools, mirrors, apertures, and lenses. In this review, we focus on recent developments in optofluidic lenses, which arguably forms the most important part of optofluidics devices. We report first on a number of general characteristics and characterization methods for optofluidics lenses and their optical performance, including aberrations and their description in terms of Zernike polynomials. Subsequently, we discuss examples of actuation methods separately for spherical optofluidic lenses and for more recent tunable aspherical lenses. Advantages and disadvantages of various actuation schemes are presented, focusing in particular on electrowetting-driven lenses and pressure-driven liquid lenses that are covered by elastomeric sheets. We discuss in particular the opportunities for detailed aberration control by using either finely controlled electric fields or specifically designed elastomeric lenses.

## 1. Introduction

Micro-optics is ubiquitous in the industry and has numerous applications in many domains, such as construction of fiber optics, mobile phone cameras, CD players, military equipment, and other consumer goods. The functionality of these devices is usually constrained by their fixed focal length and, thus, their inability to access objects present at different distances from the device. Adaptive optics offers a viable solution to this limitation. Adaptive optics can modulate the configuration of an optical surface by an external stimulus, such as an electric field, fluidic pressure, *etc*. This modulation enhances the operating range of a device by increasing the span of the device’s focal length, thus enabling it to scan objects present at varying distances. During the past few years, such miniaturized adaptive optical systems catering to the growing challenges posed by conventional optical systems have increased. The discipline is rich and diverse and is constituted by liquid crystals, deformable soft materials, and deformable liquids, such as optofluidics. Extensive reviews of various optofluidic systems, their utilities, and applications [1,2,3,4,5] are available. The advent of such optofluidic devices has provided an alternative route to re-designing and improving pristine optical systems. Such devices offer greater flexibility, supplemented by microscopic accuracy. This review focuses on a specific subset of optofluidics, namely optofluidic lenses. Such lenses have attracted considerable attention in the recent past as they are especially suited for use in adaptive optics to enhance optical performance. They have been at the epicenter of this technological innovation. A detailed review [6] is available that broadly covers the essential aspects of lens design, fabrication, and optical characterization. Optofluidic lenses come in several types: pure liquid lenses with only free liquid surfaces, liquids coated with thin elastomeric membranes, and polymeric lenses. The liquid lens literature largely deals with spherical lenses. Various techniques and tuning mechanisms have been explored to create liquid lenses, including pressure variation, thermal expansion, and electrowetting. Electrowetting (EW) has emerged as a powerful tool for manipulating the liquid–liquid interface [7,8,9,10,11]. The phenomenon has been explored to produce liquid lenses with various morphological configurations. Berge *et al.* [7] employed EW for the first time to produce spherical lenses of varying focal lengths by altering the contact angle. EW was also used by Kuiper *et al.* [8] to manipulate the liquid–liquid interface as a functioning optical lens. A similar approach was adopted by Kruipenkin *et al.* [9] to change the liquid lens curvature. Other noted mechanisms of tuning liquid lenses are hydrodynamic actuation [12],thermal stimulation [13,14,15], *etc*. These mechanisms are discussed in detail in subsequent sections. However, these driving mechanisms retain the spherical character of liquid lenses, rendering them prone to spherical aberration. The presence of such aberrations hampers the image quality, adversely affecting the optical performance. Non-spherical shapes are thus highly desirable to minimize aberrations and to improve image resolution. Consequently, several experimental techniques for inducing non-sphericity have been formulated. In a review article, Hung *et al.* [16] summarized the progress made in the fabrication techniques for producing aspherical polymeric microlenses with a high numerical aperture and the requested spot size. Roy *et al.* [17] designed and fabricated an optofluidic aspherical lens employing the Elastocapillary effect. Elastomeric lenses, an alternative to liquid lenses, with tunable astigmatism have also been reported [18]. Such lenses may have applications in correcting ocular astigmatism. Liebetraut *et al.* [19] gave an abridged account of the optical properties of various liquids used to fabricate optofluidic lenses. Another class of lenses, called gradient refractive index (GRIN) lenses, is also used in optical applications. The variation in the refractive index of the lens material is used to focus the incoming light beam. Mao *et al.* [20] reported the design and functioning of a tunable liquid gradient refractive index lens (L-GRIN lens) by optimally controlling the diffusion of calcium chloride, as the solute, in water. The concentration gradient of calcium chloride results in continuous variation in the refractive index along the concentration profile, enabling the targeted focusing of the laser beam when impinged on the mixing channel. Chen *et al.* [21] fabricated focus tunable laser induced 2D-GRIN liquid lenses. The device consists of two chromium strips for heating the enclosed lens fluid. By varying the laser intensity, thermal gradients are induced in both transverse and longitudinal directions, thereby modulating the lens shape. Such lenses offer aberration-free imaging and low actuation times, typically 200 ms. Fluidic lenses can be broadly categorized into three types of shape: spherical, aspherical, and cylindrical. In each case, we discuss the working principle and device architecture of the lens system. We further sub-classify each lens based on its actuation mechanism. Special attention will be given to two important aspects of aspherical lenses: tunability and aberration control.

This review is organized as follows: In Section 2, we provide an overview of the general characteristics of optofluidic lenses, including, in particular, a discussion on lens aberration. Section 3 focuses on the various concepts of adaptive spherical lenses, with an emphasis on EW-controlled liquid lenses. In Section 4, we discuss various approaches for controlling aberrations by generating non-spherical lens shapes. Finally, in Section 5, we discuss several optofluidics concepts in addition to lensing applications.

## 2. General Characteristics of Optofluidics Lenses

### 2.1. Liquid Lens Shapes and Actuation Principles

According to Laplace’s law, free liquid surfaces in mechanical equilibrium and in the absence of other forces display a constant mean curvature κ. The curvature is related to a pressure drop ΔP across the interface by:

(1) ΔP=2 κγ

where γ is the surface tension of the liquid. If the system is cylindrically symmetric, this results in a spherical cap shape with a radius R that is given by the inverse of the mean curvature, *i.e.*, R=1/κ. Unlike solid surfaces that have to be machined very carefully to be at the same time perfectly spherical on a global scale and perfectly smooth on a small scale of, say, λ/10 (λ: typical wavelength of visible light), liquid surfaces in equilibrium are thus perfectly spherical and perfectly smooth by the laws of physics. While some intrinsic roughness due to thermally excited fluctuating capillary waves is present in principle, the resulting roughness amplitude is no larger than O(1 nm), thanks to the strength of typical interfacial tensions (O(tens of mN/m)). In this sense, liquids are ideal materials to fabricate lenses. In addition, free liquid surfaces are perfect for adaptive optics because their refractive power, which scales as the inverse of the radius of the liquid lens, can simply be adjusted by controlling the pressure between the lens fluid and the ambient medium following Equation (1). We will denote such lenses as free interface liquid lenses, FI-LLs.

Several important caveats apply, though. Some of them are exclusively disadvantageous; others also offer opportunities for additional functionality, in particular aberration control. First, many liquids tend to evaporate, which would make any device useless. To circumvent evaporation, liquid lenses are generally designed from two immiscible liquids, such as water and oil, both contained in a sealed container. Once the ambient fluid is saturated with the lens fluid, the lens volume remains constant. Alternatively, the liquid droplet can be covered by a thin elastomeric membrane that is impermeable to the lens fluid. We will denote such lenses as elastomeric membrane liquid lenses, EM-LLs. Frequently, such membranes are made of polydimethylmethoxysilane (PDMS) with a thickness varying from several tens to a few hundred micrometers. The membranes not only suppress evaporation, they also provide a lot more stiffness to the liquid lens because the surface tension in Equation (1) is essentially replaced by the elastic tension of the membrane. One should note, though, that this comes at the expense of introducing an additional layer of a material into the optical path, which—unlike the liquid surface—is no longer automatically smooth by the laws of physics. Moreover, the thickness of such a layer also needs to be carefully controlled to be perfectly homogeneous—or laterally modulated in a perfectly controlled manner, if desired otherwise.

Second, liquid surfaces are only perfectly spherical as long as other external forces are negligible. Additional external forces such as gravity lead to deviations from the spherical shape. In the presence of gravity, Equation (1) must be extended by an additional hydrostatic pressure $\Delta P_{h}=\Delta \rho gz$, where $\Delta \rho =\rho_{l}-\rho_{a}$ is the difference between the density of the lens fluid $\rho_{l}$ and the density of the ambient fluid $\rho_{a}$, g is the gravitational acceleration. and z  is the height above the reference level, for which ΔP=2 κγ. The corresponding equation then reads:

(2) ΔP=2 κγ+Δρgz

Equation (2) states that κ=κ(z) in the presence of gravity is no longer constant. If we use the radius of curvature R of the lens as characteristic length scale and the Laplace pressure $P_{L}=\gamma /R$ as the characteristic pressure scale, Equation (2) can be rewritten in non-dimensional form as:

(3) $$\Delta \tilde{P}=\tilde{\kappa }+Bo\;\tilde{z}$$

where $Bo=\Delta \rho gR^{2}/\gamma$ is the Bond number, and $\Delta \tilde{P}=\Delta P/P_{L}$, $\tilde{\kappa }=2\;R\kappa$, and $\tilde{z}=z/R$. This implies that the contribution of gravity is negligible provided that Bo≪1 or, equivalently, $R\ll \lambda_{c}$, where $\lambda_{c}=\sqrt{\frac{\gamma }{\Delta \rho g}}$ is the capillary length. For water in air, $\lambda_{c}\approx 2.7\;\text{mm}$. To achieve spherical shapes on larger scales, it is necessary to minimize the density difference between the lens liquid and the ambient liquid. By choosing suitable oils and water and additives, it is possible to reduce Δρ to $\approx 10^{-3}\;g/{\text{cm}}^{3}$. Density matching between the lens liquid and the ambient liquid not only minimizes the effect of gravity, it also minimizes the sensitivity of the lens to ambient vibrations. A liquid lens system with perfectly density matched fluids is completely insensitive to accelerations, making such a design superior to any mechanical system with translatable solid lenses.

Like oil–water density matching, covering the lens by an elastomeric membrane also provides superior stability against both gravitational distortion and vibrations. In this case, the enhanced stability arises from the much higher membrane tension that qualitatively replaces the surface tension. It should be noted, though, that quantitatively the resulting relation between the excess pressure (or mechanical stresses applied in other manners) and the lens curvature is usually more complex because the devices are often deformed to substantial strains, leading to non-linear elastic response.

Notwithstanding this sometimes complex non-linear elastic response, the general approach to achieve tunability is the same for both FI-LLs and EM-LLs: an excess mechanical pressure or stress deforms the shape of the lens—and subsequently refraction of light from the variable lens shape provides optical tunability. If the deformation is simply achieved by increasing a hydrostatic pressure, the resulting lens will typically remain spherical. There are, however, opportunities to achieve non-spherical deformations, too. In the case of FI-LLs, arbitrary non-spherical surface shapes can be achieved if the liquid surfaces are deformed by non-homogeneous external fields, such as electric fields, which we will discuss in Section 4. For EM-LLs, asymmetric stresses and/or asymmetric thickness profiles of the membranes can provide access to non-spherical lens shapes (see also Section 4).

### 2.2. Quantification of Optical Aberrations and Lens Shapes

Optical aberrations hamper the quality of optical images. Aberrations can be quantified by analyzing the wavefront emanating from a particular optical system. The shape of the emanating wavefront is completely determined by the refractive properties of the optical systems, *i.e.*, by the shape of the lens(es) for homogeneous optical materials. For a single lens, this one-to-one correlation allows us to reconstruct the shape of the lens from the wavefront. It is customary to represent wavefronts as a superposition of Zernike polynomials, an infinite and complete set of orthonormal polynomials defined on a unit circle. The amplitude of each Zernike polynomial determines the strength of the corresponding aberration. There are several manners of numbering Zernike polynomials, depending on their number of nodes and azimuthal symmetry. Table 1 provides a list of the most common optical aberrations and the corresponding Zernike polynomials [22]. For example, spherical aberration describes the non-uniform refractive power of rotationally symmetric spherical lenses: off-axis beams are refracted more strongly than paraxial beams, resulting in a radius-dependent focal length. Non-rotationally symmetric lenses may have different curvatures in perpendicular directions, the tangential and the sagittal plane, causing astigmatic or cylindrical aberration. Imaging a point object through an astigmatic lens produces a line image. Coma results in an off-axis location of the focus of a lens.

.

The complete set of Zernike coefficients obtained with this decomposition characterizes all optical aberrations of the lens, such as. The larger the Zernike coefficient, the larger is the corresponding aberration.

The most popular and ubiquitous instrument for this type of lens characterization is the Shack–Hartmann wavefront sensor (SHWS) [23]. A SHWS allows us to reconstruct the distortion of a wavefront from the deflections of the focus positions of an array of microlenses on a CCD (charge coupled device) sensor. The 3D wavefront is re-constructed from these focus positions using inbuilt numerical algorithms. From the aberrations measured by a SHWS, it is also possible to calculate the surface profile of the lens in three dimensions, as shown by Li *et al.* [24] for liquid microlenses.

Throughout this review, we will pay particular attention to cylindrically symmetric lenses, for which primary spherical aberrations are the most important aberration. The ideal shape of a cylindrically symmetric lens transforming light emitted from a point source into plane waves is a hyperbola with an eccentricity $e=n_{l}/n_{a}>1$, where $n_{l}$ and $n_{a}$ are the refractive indices of the lens material and of the ambient medium. With ideal lens shapes being derived from conic sections, it has become customary to characterize the shape of general rotationally symmetric aspherical lenses by the equation:

(4) $$z\left(r\right)=\frac{r^{2}}{R\left(1+\sqrt{1-\left(1+K\right)\frac{r^{2}}{R^{2}}}\right)}+\alpha_{1}r^{2}+\alpha_{2}r^{4}+\alpha_{3}r^{6}+...$$

where r is the radial coordinate and z the axial position of the lens surface. The first term in Equation (4) describes the ideal conic section with the conic constant $K=-e^{2}$ and the radius of the lens apex R. The other terms describe deviations from the ideal conical shape with ‘aspherical coefficients’ $\alpha_{i}$ of the symmetric algebraic terms in r. Conic lenses are a subset of aspherical lenses, with all $\alpha_{i}=0$. The eccentricity is zero for spherical lenses, unity for parabolics, between zero and unity for elliptical lens, and greater than unity for hyperbolic lenses. For K=0 and $\alpha_{i}=0$, the equation is reduced to that of a sphere signifying spherical lenses. These lenses have spherical aberrations, which implies that off axis marginal rays entering the lens are more strongly refracted than paraxial rays. The difference between paraxial and marginal focal lengths is called longitudinal spherical aberration (LSA). It increases with the numerical aperture of the lens. It is difficult to completely suppress and eliminate the spherical aberration even after standard remedial measures, such as polishing, for improving the optical quality of the lens.

In conventional optical systems multiple optical elements are employed to compensate optical aberrations. Upon adjusting the focus position or zoom factor of an optical system, the optical elements have to be translated longitudinally with respect to each other to guarantee optimum imaging quality. This requires a complex design and complex mechanics of optical systems. Adaptive optics with tunable optical aberrations such as asphericity offers the promise of simultaneous small aberrations and simple optical system design.

The easiest approach to assess the optical performance of the adaptive lenses is to measure the shape of the lens and to calculate the resulting refraction properties of the system using ray tracing procedures as implemented in many software packages, such as for instance Zemax. Geometrical properties of captured optical side view images of liquid lenses such as conic constants and eccentricity can be easily extracted and imported to optical simulation platforms like Zemax for evaluating their optical properties. In addition to simple optical imaging of lens shapes, more advanced techniques such as phase-shift interferometry and holography can be used to characterize lens shapes with high accuracy, thereby enabling detailed computations of the resulting optical aberrations. Interferometry is regularly used for measuring optical aberrations present in liquid lenses (see e.g., [25]). Interferometers are often employed for detecting optical aberrations of the lenses. Santiago *et al.* [25] employed interferometry for computing spherical aberration present in hydro-pneumatically tunable variable focus liquid lens.

### 2.3. Quantification of Optical Performance

The performance of an optical system is commonly characterized by the modulation transfer function (MTF) and the root mean square (RMS) spot size. The MTF and the spot size are alternative, complementary measures for characterizing the quality of an optical system. The MTF is a numerical measure of the transfer of intensity modulation or contrast from the object to the image. It represents how accurately intensity gradients and details of an object are mapped by an optical system to the image plane. Standard sinusoidal resolution targets are used to measure the MTF in laboratory experiments. They consist of multiple black and white stripes with variable spatial frequencies. Thus, imaging a single target allows us to determine the resolution of an imaging system at a number of spatial frequencies. When an object is imaged through a lens, a higher resolution is obtained for an object with lower spatial frequency. However, as the spatial frequency increases, the contrast degrades, the image is blurred, and the intensity modulation is reduced. While diffraction determines the ultimate limit of modulation transfer, aberrations of non-optimized optical systems frequently lead to a degradation of the image quality for lower spatial frequencies than expected. The closer the MTF of an optical system is to the ideal diffraction limited curve, the better the optical performance of the system [26].

The RMS spot size, on the other hand, describes the distribution of rays on the image plane upon illuminating the full back aperture of the system. The system is diffraction limited if the image spot falls within the confines of the Airy disc. Overall, the MTF provides the more quantitative and complete characterization of the lens properties. Even if all rays in a spot diagram fall within the Airy disc, this is not sufficient to guarantee optimum optical performance, as shown by the deviations in the MTF curves.

In diffraction limited systems, the RMS wavefront error is another valuable measure to quantify deviations from perfect imaging. For optical systems with considerable optical aberrations, the peak to valley (P–V) error can also be of interest. According to the Rayleigh criterion, an optical system is considered optically sound if the P–V error is <λ/4. However, the P–V error only measures the difference between the maximum and minimum values, while the RMS wavefront error illustrates a more holistic picture of the wavefront map by accounting for all crests and troughs. Strehl’s ratio is another standard norm used to analyze optical quality. This ratio is defined as the ratio of the peak intensity of an optical system with aberrations to the diffraction-limited aplanatic optical system without aberrations. A high Strehl’s ratio signifies improved optical performance. This ratio is particularly important for characterizing diffraction-limited systems. Aspherical lenses are in demand because of their superior optical properties: improved aberration control, diffraction-limited MTF, and a higher Strehl’s ratio. Krogmann *et al.* [27] compared the optical performance of solid fixed-focus microlenses against the tunable liquid lenses. By comparing RMS wavefront error values these authors concluded that liquid microlenses and solid microlenses achieve comparable optical quality.

### 2.4. Materials and Design Considerations

The functionality and lifetime of optofluidic lenses are often limited by the fabrication material and other design constraints. For instance, membrane-encapsulated lenses suffer from reduced lifetime due to repeated expansion and contraction of the membranes under the application of pressure stimulus. Additionally, unlike pure liquid lenses which have smooth optical surface, membranes require further surface characterization. Similarly, for EW-lenses, Teflon-coated hydrophobic substrates are prone to degradation during regular operation, in particular if water is used as a conductive fluid. Alternative non-aqueous conductive fluids such as ethylene glycol offer much longer lifetimes for EW-lenses. Furthermore, suitable ambient oils as well as the use of AC voltage of sufficiently high frequencies helps to minimize contact angle hysteresis [28].

In addition to the aberrations discussed above, chromatic aberrations also degrade the quality of optical images for conventional color imaging applications. However, specific lens fluids with low dispersion are available that minimize the effect even in the absence of specific corrections. Like for conventional optics, however, it is also possible to compensate for chromatic aberrations beyond material optimization by introducing multi-component lens systems that compensate for each other’s aberrations. Waibel *et al.* [29] demonstrated such a system composed of different liquids and membranes.

Next to tuning range and aberrations, actuation speed is an important characteristic for adaptive lenses. The maximum speed can depend either on the actuation mechanism or on the intrinsic properties of the deformable lens. Except for the case of thermally driven lenses, which typically involve long thermal relaxation time constants of the entire device, the speed of pressure actuators (e.g., piezos) or electric fields is typically very fast. In this case, the response time of the fluid is usually the limiting factor. A basic estimate of the response time can therefore be obtained by considering the eigenmodes a liquid droplet, determined by the balance of surface tension and inertia. A free droplet in air has a discrete spectrum of eigenmodes with eigenfrequencies given by:

(5) $$\omega_{n}=\sqrt{\frac{\gamma }{\rho_{l}R^{3}}}\;\sqrt{n\left(n-1\right)\left(n+2\right)}$$

as first calculated by Rayleigh in the 19th century. For a millimeter-sized drop of water, this results in an eigenfrequency of approximately 65 Hz for the lowest eigenmode (n=2). For sessile drops on a solid surface embedded in an ambient oil of finite density and viscosity, these frequencies are slightly reduced. For instance, for the same sized water drop in silicone oil with a viscosity of 5 mPas, the lowest resonance frequency is reduced to 55 Hz [30]. (For higher eigenmodes, the frequency shift is less pronounced.) Exciting the liquid lens at frequencies close to the lowest eigenfrequency generally leads to substantial distortions of the lens surface during actuation, followed by an oscillatory ring-down of the excited eigenmodes. While the addition of viscosity modifiers to the liquid can dampen undesired oscillations following a step-actuation [7], it is generally reasonable to assume that lenses can only be operated up to some critical frequency somewhat below the lowest eigenfrequency. Actuation frequencies beyond a few tens of Hz can thus only be obtained by reducing the lens aperture to sub-millimeter scales. An exception to this rule is lenses that are operated in an oscillatory mode, in which the focus is continuously modulated between a maximum and a minimum. In this case, actuation frequencies with reasonable optical image quality of up to 3 kHz have been demonstrated experimentally [11,31].

Next, we classify lenses based on their shapes and further sub-classify the lenses according to the respective driving stimulus.

## 3. Spherical Lenses

Liquid spherical drops are ubiquitous in nature. Due to the mismatch in the refractive index between a liquid drop and its ambient fluid, the liquid drop can function as an optical lens. Optical characteristics, such as focal length, of such liquid lenses are determined by the drop configuration and material composition. Thus, by tuning the two parameters, the meniscus curvature and the drop-ambient material phase, one can manipulate the optical properties of liquid lenses. The morphological transition of the liquid–liquid interface can be induced by an umpteen number of driving mechanisms. Other methodologies for fabricating lenses include thermally actuated lenses [13,14,15], pneumatically driven lenses [32], fluidic pressure lenses [12], membrane-encapsulated fluidic lenses [33], electrochemically activated lenses [34], stimuli-responsive hydrogels [35,36], harmonically driven lenses [31], electrowetting lenses [7,8,9,10,11], *etc*. Such adaptive liquid microlenses have an adjustable focus, and their response time varies from milliseconds to tens of seconds. Based on the actuation mechanism, these microlenses can be further classified into the following types.

### 3.1. Thermally Driven Lenses

In thermally actuated lenses, the refractive power of an enclosed optical liquid is altered by using thermal expansion. Lee *et al.* [13] enclosed optical fluid in the conducting ring attached to external heaters. As power is supplied, the induced temperature gradient causes the liquid to expand, consequently increasing its volume and surface area. This, in turn, changes the radius of the curvature and, thus, the focal length.

**Figure 1:**
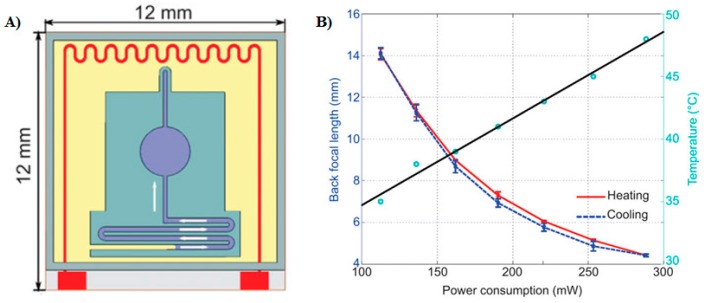
(**A**) Enclosed silicon oil (light purple) in PMMA membrane is heated by raising the temperature of the ambient air (in yellow). Red contact pads represent the heat source. (**B**) Back focal length (mm) of the lens *vs.* temperature during heating and cooling ramps. The solid black line denotes the linear fit of the variation in the power consumption (blue circles) against the increasing temperature. Reprinted by permission from Macmillan Publishers Ltd.: *Nature LSA*, Zhang *et al.* [14], copyright 2013.

In a recent study reported by Zhang *et al.* [14], shown in Figure 1A, the surface area of the silicon oil, trapped in the polyacrylate membrane, is increased by increasing the temperature of the ambient air. As the surrounding air expands, it displaces the silicon oil present in the vent connected to a deformable polyacrylate membrane. This causes the enclosed silicon oil to push the flexible membrane radially outward, thus increasing its surface area. The lens shows reasonably good reversible behavior with negligible hysteresis, less than 0.5 mm in focal length, when subjected to heating and cooling cycles. As depicted in Figure 1B, the heating and cooling cycle curves overlap as the temperature is increased and decreased, respectively. The voltage requirement for device operation is around 7.5 V. To further quantify the imaging performance, the MTF was measured at different focal lengths, corresponding to different numerical apertures. The best MTF performance was achieved at the longest focal length. The MTF is degraded when the focal length decreases or the numerical aperture increases. This is contrary to the expected outcome. It occurs because of irregular aspherical deformations as the temperature is raised. Schuhladen *et al.* [37] employed thermally actuated liquid crystal elastomers to fabricate an Iris-like tunable aperture, mimicking the human eye. Thermally tunable lenses have a serious disadvantage: poor response time, which makes them non-suitable for applications that require fast-switching. Moreover, frequently subjecting the thermally actuated lens device to heating and cooling cycles damages the mechanical structure of the device.

### 3.2. Pneumatically Driven Lenses

**Figure 2:**
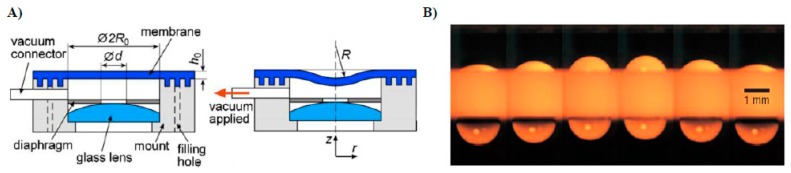
(**A**) Oscillating liquid lens under a forcing amplitude of 5.5 Pa. Reprinted from Kyle, C.; Fainman, Y.; Groisman, A. Pneumatically actuated adaptive lenses with millisecond response time. *Appl Phys Lett* **2007**, *91*, 171111. With the permission of AIP Publishing. (**B**) Composite optical system of a glass lens (light blue) and a planar elastomeric membrane (dark blue) in an undeformed state provided with a vacuum connector. Deformed membrane under applied vacuum. Reprinted by permission from Macmillan Publishers Ltd.: *Nature Photonics*, Lopez and Hirsa [31], copyright 2008.

Liquid lenses are also often constructed with optical liquid enclosed by a membrane. Such lenses are usually operated by applying fluidic pressure or pneumatic actuation or by mechanical stress. Ren *et al.* [38] demonstrated a mechanically actuated focus tunable liquid lens, by enclosing the liquid under a deformable elastic membrane controlled by a servo motor. Werber and Zappe [39] fabricated tunable microfluidic microlenses activated by fluidic pressure. These membrane-encapsulated fluidic lenses often use PDMS membranes to enclose the lens fluid. PDMS is preferred because of its ease of machinability. Figure 2A depicts a pneumatically actuated lens [32]. The optical refractive element is an elastomeric flexible membrane (in dark blue). It is integrated with a mounted camera lens. The diaphragm restricts the amount of light eventually received by the membrane lens. As the vacuum is switched on, the membrane deforms and bends inward, adopting an aspherical configuration. The deformed membrane can act as an optical lens. The dependence of the refractive power of the compound lens system on the applied pressure was further investigated, and the optical system was characterized by imaging black and white strips on a charged couple device (CCD). Such lenses have very short response times, typically a few milliseconds. However, the lens configuration is not very well defined. Consequently, membrane shapes are not very amenable to standard characterization techniques. External pressure actuators are then employed to change the lens curvature. Piezoelectric, electromagnetic, and thermal actuators have been designed for this purpose.

Choi *et al.* [40] proposed a magnetically actuated fluidic lens. The optical liquid is entrapped by a double-sided PDMS membrane. Varying the distance between the membrane interfaces can be used to tune the focal length of the doublet lens. In addition, the proposed double-sided lens system compensates for the spherical aberration. Reichelt and Zappe [33] outlined a design for a spherically corrected, achromatic, variable focal length lens. After modeling and optimizing the proposed design on Zemax, the researchers postulated that choosing the appropriate optical liquids of a composite lens system, composed of multiple membrane fabricated lenses, can significantly mitigate chromatic aberration and primary spherical aberration. The lens can be created by pneumatic actuation or by electrowetting. Chronis *et al.* synthesized a microfluidic network of PDMS-sheathed liquid oil microlenses [41]. The lenses were tuned in tandem in sync by stimulating them pneumatically. Varying the pneumatic pressure can change the focal lengths of the microlenses. Beadie *et al.* [42] employed compressive mechanical stress to design a composite tunable polymer lens. The lens system consists of a hard PMMA plano convex lens as a backing plate with a PDMS cured plano-convex lens rigidly stacked on the planar surface of the PMMA lens. The researchers observed that the focal length of the composite lens could be changed by a factor of 1.9 mm with applied compression of 1.3 mm. Pneumatic actuation of optical liquids requires membrane encapsulation. This is a disadvantage as it poses an additional demand on membrane characterization; for example, the membrane has to be smooth, with well-controlled RMS roughness. Moreover, membranes are often fragile and are prone to wearing off due to regular usage. This calls for their regular periodical replacement.

A completely different approach was developed by Lopez and Hirsa [31] by fabricating fast-focusing, harmonically driven liquid lenses. Instead of a quasi-static operation, these authors actuated liquid lenses in a dynamic mode by continuously modulating the lens shape at an elevated frequency. The experimental setup shown in Figure 2B consists of a 1.82 mm thick Teflon-coated plate, cylindrically drilled along the thickness. The coupled lens system is formed by water droplets pinned on either side of the Teflon plate along the two apertures, each 1.68 mm in diameter. The system is excited by a pressure source at a frequency of 49 Hz. The fast focusing ability of the lens system is confirmed by optically imaging a standard resolution target. Focal length at any given time is calculated with Snell’s law. As the lens optical system is composed of two convex lenses, it has the ability to rectify the spherical aberration. This is due to the fact that aberration due to one lens can be nullified by equivalent aberration of opposite magnitude by the other lens. However, since the lens is operated in ambient air rather than in another liquid, it remains vulnerable to evaporation. Hence, the lens meniscus cannot sustain its initial topography and suffers from poor shelf life. Due to the absence of any ambient liquid, the effect of gravity, which induces flatness in the lens profiles, cannot be neglected.

### 3.3. Stimuli-Responsive Lenses

**Figure 3:**
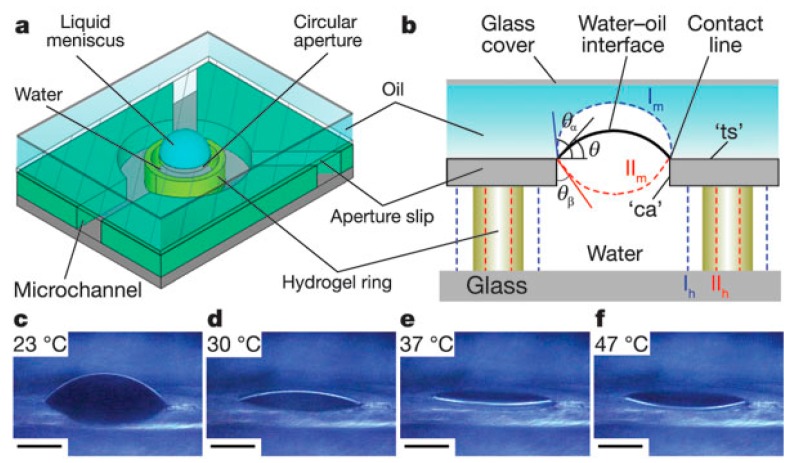
(**a**) Experimental setup; (**b**) liquid meniscus in a circular aperture via the pinned contact line formed by the top hydrophobic surface, represented by ts and hydrophilic bottom substrate and sidewalls. The contact angles ca are modulated by an entrapped stimuli-responsive hydrogel. Dashed blue lines represent a divergent lens while red dashed lines correspond to the convergent lens profile. (**c**–**f**) Morphology of the water–oil interface at different temperatures. Reprinted by permission from Macmillan Publishers Ltd.: *Nature*, Dong *et al.* [35], copyright 2006.

Stimuli-responsive hydrogels can also be employed as a viable tool for manipulating the curvature of the water–oil interface to produce variable focal length microlenses. In the system described by Dong *et al.* [35], shown in Figure 3, a hydrogel ring is sandwiched between the two plates; the top plate has an opening.

The microfluidic channel, shown in Figure 3, is filled with water. The hydrogel ring is surrounded by a polymer jacket to constrain the expansion or contraction of the hydrogel. Water is then loaded into the space, enclosing the hydrogel ring and the plates, followed by oil as an ambient fluid. The hydrogel expands or shrinks in response to the external stimulus, thus changing the volume of the enclosed water, which alters the curvature of the oil–water interface from flat to some arbitrary spherical shape that corresponds to a specific Laplace pressure. The contact line is firmly pinned by the hydrophobic–hydrophilic aperture boundary. The change in the curvature of the interface, which can be translated into respective focal lengths, depends on the strength of the stimulus. The external stimuli can be temperature or pH. The liquid meniscus grows at a lower temperature. This is because the volume of water lost due to absorption by the hydrogel is less than the expanded volume of the hydrogel itself. Conversely, at higher temperatures, the meniscus shrinks as the amount of water expelled is considerably less than the contracted volume of the hydrogel ring. However, these lenses suffer from a long response time, typically 12–15 s. In addition, the spherical shape of the droplet is retained, leaving such lenses vulnerable to optical aberrations. Zeng *et al.* [43] improved the response time by using infrared actuation of a light-responsive hydrogel. For a broader field of view, Zhu *et al.* [36] fabricated a microlens array on a curved hemispherical glass surface. A thermo-responsive hydrogel was employed to regulate the curvature of the water–silicone oil meniscus. The curvilinear configuration has a significant advantage over the planar surface by offering a much larger field of view.

Miccio *et al.* [44] experimentally demonstrated the potential application of RBCs as a tunable liquid bio-lens. The focal length can be tuned by altering the osmolarity of the ambient medium. Imaging through the RBCs array helps in diagnosing the blood deficiencies. This was ascertained by dynamic wavefront characterization the RBCs lens array, further corroborated by numerical modelling. Any blood disorder can be readily identified by the deviation of focal spots as observed through the RBCs of diseased blood samples compared to normal healthy cases. However, the development of such bio-lenses is still in its infancy and requires more attention. The lenses suffer from a long response time of typically 10 s because of a delay between the variation of the osmolarity of the ambient medium and the response of the lens.

### 3.4. Electrically Driven Lenses

**Figure 4:**
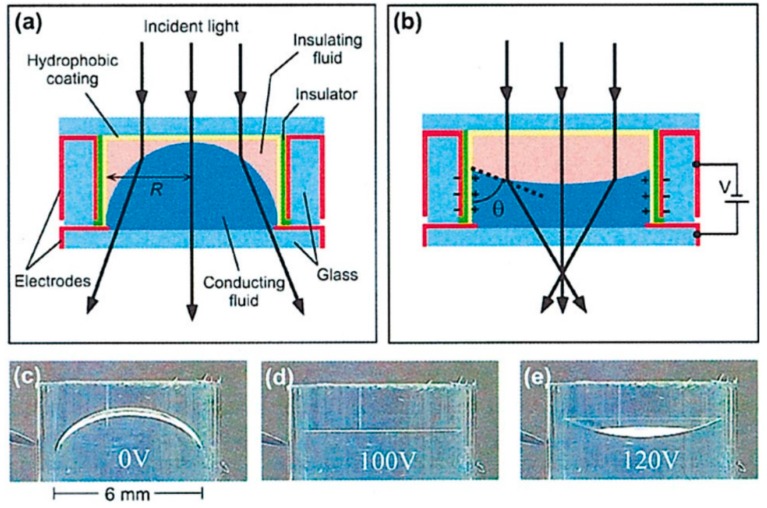
(**a**) Liquid lens system in a cylindrical housing. The red surface denotes conducting electrodes, followed by insulator coating (in green) with further deposition of hydrophobic coating. Conducting fluid (dark blue) forms a divergent lens at zero voltage. (**b**) Application of voltage ‘V’ modulates the meniscus shape to a convergent lens profile. (**c**–**e**) Topological change in lens shape from initially divergent spherical meniscus at zero voltage to a flat interface at 100 V and subsequently to convergent lens at 120 V. Reprinted from Kuiper, S.; Hendriks, B.H.W. Variable-focus liquid lens for miniature cameras. *Appl. Phys. Lett.* **2004**, *85*, 1128–1130. With the permission of AIP Publishing.

Liquid manipulation by electric field [45,46] is also widely investigated because of its paramount importance in domains such as adaptive optics, optical switching, displays, *etc*. EW lenses [7,8,9,10,11] are fast, demonstrating excellent switching speed, and offer a good degree of tunability in focal length. They offer higher flexibility in design without any mechanically moving parts. The concept has also been explored for miniaturized systems. However, in most of the studies reported so far, the spherical shape of the lens meniscus is restored, and thus these lenses suffer from spherical aberrations, which, in turn, decrease their optical performance. EW [47] modulates the contact angles between the fluid and the substrate on which the liquid drop rests. The general EW equation is:

(6) $$\text{cos}\theta ={\text{cos}\theta }_{0}+\frac{\epsilon \epsilon_{0}}{2d\gamma }U^{2}$$

where $\theta_{0}$ is the Young angle at zero voltage, θ is the contact angle under the influence of applied voltage U, d is the thickness of the dielectric, γ is the liquid–liquid interfacial tension, ϵ is the electrical permittivity of the droplet fluid, and $\epsilon_{0}$ is the permittivity of the free space. Thus, applying a potential U between the droplet and the dielectric can alter the contact angle between the drop and the substrate on which the droplet rests. One challenge is that the droplet should remain at its optical center, thus precluding any unwanted optical distortions. This has been successfully achieved by adopting various self-centered lens designs. In the system reported by Kuiper and Hendriks [8], depicted in Figure 4, the liquid–liquid interface is modulated by EW actuation on the sidewalls. The sidewall consists of embedded electrodes coated with a hydrophobic dielectric layer, capable of electrowetting modulation. The entire system is integrated in a cylindrical housing. Conducting aqueous solution is used in the drop phase, while insulating fluid is employed as an ambient liquid. The presence of ambient fluid also arrests the evaporation of the drop fluid. Due to the density-matched system, the Bond number is sufficiently low, and consequently the meniscus shape is not measurably affected by the gravitational forces. As the voltage is applied, the interface adopts convex and concave shapes in a tunable fashion and attains a flat interface intermittently. This is shown in Figure 4. The effect of fluid viscosities on the switching speed of the lenses was also investigated. Optimized performance is obtained for critically damped systems, e.g., by adding suitable viscosity modifiers such as PEO (polyethylene oxide) [7,8]. These lens systems are devoid of meniscus oscillations and hence focusing speed is not sacrificed.

**Figure 5:**
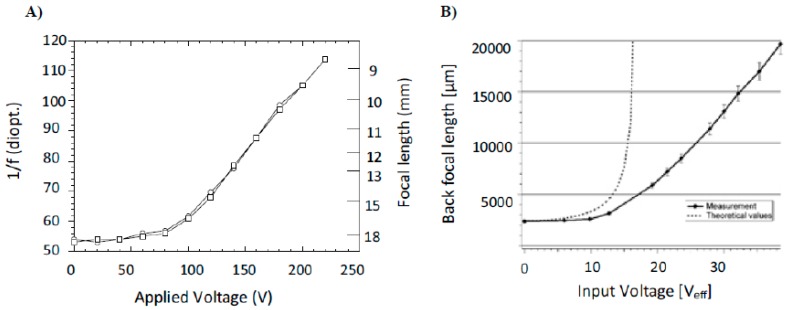
(**A**) Variation in focal length (in mm) and power of lens (inverse of focal length) with the applied voltage. The two curves superimpose as the voltage is increased and decreased, respectively. With kind permission of the *European Physical Journal E* (EPJE). Adapted from Berge, B.; Peseux, J. Variable focal lens controlled by an external voltage: An application of electrowetting. *Eur. Phys. J. E* **2000**, *3*, 159–163. (**B**) Measured and theoretical values of back focal length (in µm) *vs.* applied electrowetting voltage (in volts). With kind permission of The IOP Publishing material. Adapted from Krogmann, F.; Monch, W.; Zappe, H. A MEMS-based variable micro-lens system. *J. Opt. A Pure Appl. Opt.* **2006**, *8*, S330–S336.

In the pioneering work by Berge *et al.* [7], EW lenses were manufactured with wettability gradients. The gradient is induced by using dielectrics of variable thicknesses. In addition, the focal distance of such a lens can be tuned in a reversible manner as voltage is applied and released back and forth. The first plot of Figure 5A shows the variation in power (in diopters) for the applied voltage of a 6 mm diameter lens, filled with α − chlonaphthalene as the insulating fluid and the aqueous solution of sodium sulfate as the ambient fluid. Superimposition of the two curves, corresponding to forward and backward cycles, respectively, clearly depicts the very reversible nature of the liquid lens. Electrowetting was further explored by Krogman *et al.* [48], employing trapezoidal grooves as sidewalls for EW. The technique was particularly successful in self-centering the liquid lens along its optical axis. Figure 5B depicts the variation in the focal length against the applied electrowetting voltage. They applied voltage to tune the focal length from 2.3 mm at 0 V to a flat interface with infinite focal length at 45 V. Lee *et al.* [49] carried out numerical simulations and studied the evolving meniscus shapes with an application of EW, corroborating the experimental findings of Krogman *et al.* [48]. They also showed that spherical aberration can be essentially eliminated by applying an electric field on the spherical liquid meniscus entrapped in the groove. Characterization of the dynamic mechanical stability of liquid-filled lenses was also studied by Yu *et al.* [50].

**Figure 6:**
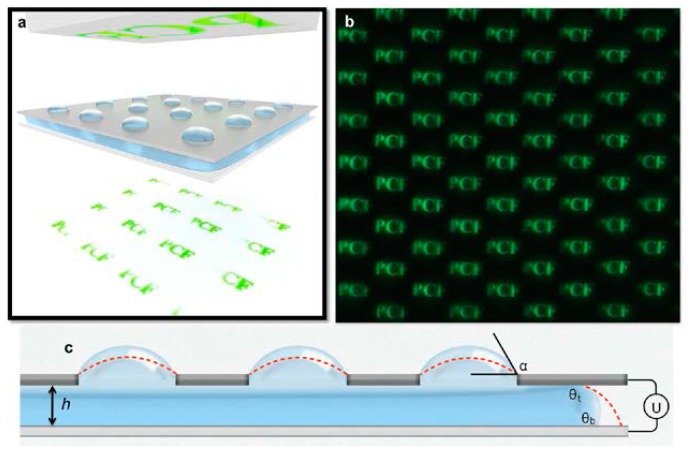
(**a**) Schematic representation of a microlens array. The PCF logo imaged from the top. (**b**) Array of PCF images formed by individual microlenses. (**c**) Side view of the microlens array. Application of voltage U modulates the contact angle of the sandwiched liquid on the bottom substrate, consequently altering the lens angle α, which in turn changes the curvature and the focal length of the pinned droplet. With kind permission of the Optical Society of America (OSA). Adapted from Murade, C.U.; van der Ende, D.; Mugele, F. High speed adaptive liquid microlens array. *Opt. Express* **2012**, *20*, 18180–18187.

The concept of creating a single EW-modulated microlens can be further extended to create a microlens array. The methodology was demonstrated by Murade *et al.* [11], as shown in Figure 6. The experiment consisted of two plates with an aperture in the top plate and a bottom plate capable of electrowetting modulation. By using the pinned contact lines, the water droplet is entrapped in the aperture and is sandwiched between the two plates. Pressure in the droplet is regulated by applying the voltage between the two plates. Hysteresis on the bottom plate is reduced by soaking it in the silicone oil. The reduced hysteresis causes frictionless movement of the contact line on the bottom plate. Hysteresis can be further suppressed by employing high AC frequency [28] for efficient depinning of the contact lines. This essentially prevents the contact line of the droplet being trapped in the defects. As the voltage is applied, the contact angle on the bottom substrate decreases, consequently lowering the pressure in the entrapped droplet. This leads to an increased radius of curvature and, thus, enhanced focal length. Parallelization into the microlens array is realized by integrating multiple apertures. This approach requires a single actuation electrode and, thus, precludes the use of a dedicated addressable electrode for each individual spherical lens. The focal length of all microlenses can be tuned simultaneously by regulating the pressure in a single reservoir droplet. The optical performance of the device is demonstrated by synchronous modulation of focal lengths beyond 1 kHz. The device has additional applications in integral imaging and 3D imaging. Replacing the conductive lens fluid with a dielectric fluid results in a dielectric lens system [51,52,53]. Due to the non-zero electric field across the dielectric lens fluid, it experiences additional bulk force due to the gradient in the electric field.

Grilli *et al.* [54] fabricated a microlens array using polar electric crystal of LiNbO_3_. This was achieved by depositing a thin oil film on square array of hexagonal LiNbO_3_ crystals. The periodically poled crystal substrates are electrically actuated by the pyro-electric effect, by subjecting the crystals to subsequent heating and cooling cycles, altering the oil topography and consequently modifying the surface tension, thereby producing a microlens array. The focal length is measured from the phase profiles extracted by interferometric measurements. The configuration is electrode-less, does not require any external electrical circuits, and is devoid of any mechanically moving parts. A similar electrode-less arrangement was utilized by Miccio *et al.* [55] for constructing tunable liquid micro-lens array. The pyro-electric activation of polar dielectric crystals generated two different regimes: separated lens regime (SLR) and wave-like lens regime (WLR). The lens aberrations were computed by analyzing the wavefront maps.

Gorman *et al.* [56] demonstrated the principle of controlling the lens shape with electrochemical desorption. The surface properties of the gold surface are manipulated by applying potential across the self-assembled monolayers. Focal length can also be tuned by electrochemically modulating the surface tension of the lens liquid [34]. The electrochemical activation is attained by applying the voltage across the lens medium. Lee *et al.* [57] propounded the construction of variable focus, tunable liquid lens using a deformable PDMS membrane. Electromagnetic stimulation was used to apply pressure on the membrane, thus changing the focal length of the lens. Electroactive polymers (EAPs) are also promising candidates because of their low response time and high flexibility. Choi *et al.* [58] exploited the EAP actuation to generate fluidic pressure that can be directed to modulate the shape of a transparent elastomer membrane. By modulating the strength of EAP actuation, varying degrees of change in the focal length of the lens membrane can be produced.

Liquid lens technology also involves designing lithographically structured electrodes to constrain droplet movement. In a study reported by Kruipenkin *et al.* [9], the droplet position can be altered by applying bias voltage across specific electrodes. It can also be constrained at the center by applying equal voltage on the electrode. These electrodes induce a spatially heterogeneous electric field, thus intensifying the field strength at specified locations and in a particular direction, eventually driving the material flow along the intensified field path. Liu *et al.* [10] employed double-ring planar electrodes for fabricating electrowetting actuated liquid lenses. Xu *et al.* [59] designed a dielectrically actuated lens placed on a well-shaped bottom electrode with a top planar electrode. This arrangement provides automatic self-centering of the dielectric liquid droplet trapped in the well-shaped electrode.

## 4. Non-Spherical Lenses

**Figure 7:**
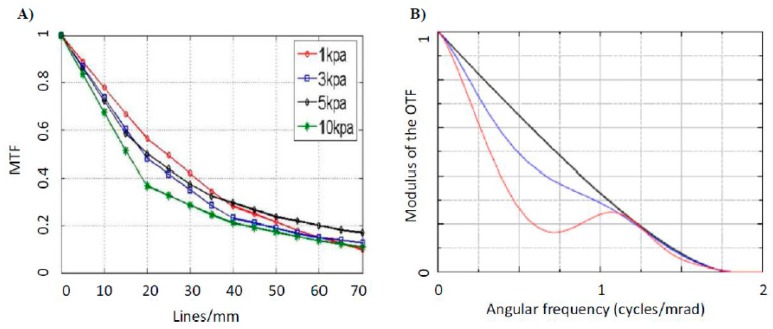
(**A**) Experimentally measured MTF curves of tunable liquid lenses under increasing pneumatic pressure from 1 to 10 kPa. With kind permission of the Optical Society of America (OSA). Adapted from Zhang, W.; Aljasem, K.; Zappe, H.; Seifert, A. Highly flexible mtf measurement system for tunable micro lenses. *Opt Express* 2010, *18*, 12458–12469. (**B**) The MTF of the astigmatic lens along two axes: tangential (T) and sagittal (S) *vs.* frequency. The black curve signifies the diffraction-limited MTF. With kind permission of the Optical Society of America (OSA). Adapted from Lima, N.C.; Cavalli, A.; Mishra, K.; Mugele, F. Numerical simulation of astigmatic liquid lenses tuned by a stripe electrode. *Opt. Express* **2016**, *24*, 4210–4220.

Non-spherical lenses can be broadly categorized into two types: aspherical lenses and cylindrical lenses. Unlike spherical lenses that have a fixed unique curvature, these lenses have a variable curvature along the surface profile. Out of all possible aberrations, spherical aberration is the most difficult to eliminate. Due to the varying curvature, aspherical lenses can overcome spherical aberration. Moreover, liquid aspherical lenses are also tunable in focal length, apart from correcting spherical aberration. They can be used to replace multiple spherical lenses in any specific optical system, thus reducing the complexity and weight of the overall optical equipment. Another class of non-spherical lenses is called astigmatic or cylindrical lenses. Astigmatism occurs because of rotational asymmetry between two principal axes perpendicular to each other, namely, tangential and meridional. Thus, such an optical surface has multiple foci. The degree of astigmatism depends on the separation between the two focal points. Spherical lenses, which are rotationally symmetric, have a single radius of curvature and thus do not exhibit astigmatic properties, as any beam of light impinged on a spherical lens will converge or appear to converge to the same focal point. However, subjecting elastomeric or fluidic lenses to asymmetric strain imparts asymmetry in the rotational configuration. Consequently, the curvature of the lens along the two axes, tangential and meridional, is different. Thus, instead of a single focus, the strained astigmatic lens possesses two astigmatic foci. This strain can be induced by a number of other mechanisms. Imaging a point through cylindrical lens yields a line. This is further illustrated by the MTF plots of the spherical and astigmatic lenses depicted in Figure 7. Zhang *et al.* [60] devised an experimental technique to measure the MTF of a PDMS-enclosed liquid lens at different pneumatic pressures. As shown in Figure 7A, as the applied pressure is increased from 1 to 10 kPa, the MTF degrades. This can be attributed to the increase in optical aberrations at higher pressures. Similarly, in the study of astigmatic lenses reported by Lima *et al.* [61], astigmatism becomes more pronounced, as the voltage is applied between the liquid meniscus and the stripe electrode. The two blue curves, shown in Figure 7B, represent the MTFs along two axes, tangential (T) and sagittal (S), while the black curve represents the diffraction-limited MTF. The higher the divergence between the two curves (T and S), the larger the degree of astigmatism present in any optical system.

In subsequent sections, we shall explore the various driving mechanisms for achieving the requisite asphericity and astigmatism. Next, we classify non-spherical lenses based on their driving mechanism.

### 4.1. Electrically Driven Aspherical and Cylindrical Lenses

Oh *et al.* [62] showed that the electric field is a potent tool for effectively switching a liquid micro-meniscus pinned by a circular aperture. As the potential is applied between the conducting droplet and the bottom electrode, kept at distance *h* from the aperture plate, the droplet is transformed into different aspherical shapes and assumes morphologies ranging from a parabola to a hyperbola and then to higher degrees of aspherical configurations. These shapes can be characterized by profile extraction techniques followed by fitting protocols, from which the nature of the conic section can be inferred. The meniscus acquires aspherical shapes of varying eccentricities as the voltage increases from 0 to 1700 V, starting from an initially flat interface. The switchability behavior depends on the aspect ratio of the pillars. The equilibrium surface profiles are calculated by balancing the electrical Maxwell stress and the Laplace pressure determined by:

(7) $$\Delta P_{h}=2\gamma \;\kappa (r)-\Pi_{el}(r)$$

where $\Pi_{el}\left(r\right)=\;\frac{{\varepsilon \varepsilon }_{0}}{2}E{\left(r\right)}^{2}$ is the electric Maxwell stress. (${\varepsilon \varepsilon }_{0}:$ dielectric permittivity of the oil). Considerable effort has been expended to fabricate aspherical polymeric lenses with the application of an electric field. Zhan *et al.* [63] fabricated polymeric aspherical lenses by applying an electric field to a droplet of SU-8 25 resting on a planar electrode surface. Under finite voltage, the polymeric material experiences Maxwell stress and acquires an aspherical shape. The materials are then subjected to UV curing for solidification. The captured droplet image profiles are fitted with the standard aspherical lens equation. The conic constant and the curvature of the droplet at the apex are extracted from the fitted profile. Droplet morphology is distorted by the application of voltage from an initially spherical shape at zero voltage to higher degree conics at a finite voltage. Beyond a critical voltage of 5150 V, the electrostatic force exceeds the restoring Laplace pressure, and the droplet becomes unstable. The spatial resolution of the cured aspherical lens, as given by the Rayleigh criterion, is 1.325 μm, which is smaller than the diameter of the airy disc. The sagittal and tangential MTF curves approach the diffraction-limited MTF. Further, the lens has a Strehl’s ratio of 0.742. The calculated optical metrics indicate that the lens has significantly reduced aberrations. However, these lenses are not tunable. During fabrication, the polymer responds slowly to the applied electric field to assume the desired shape. Once cured with UV light, however, the shape and thus the optical properties of the lenses are fixed like for any ordinary solid lens. Polymeric lenses require further characterization of surface smoothness. Kuo *et al.* [64] fabricated a tunable SU-8 negative photoresist aspherical microlens array by electrostatically pulling the SU-8 microdrops. The aspherical drops are further UV cured. The asphericity of the conical shapes is controlled by the applied voltage.

**Figure 8:**
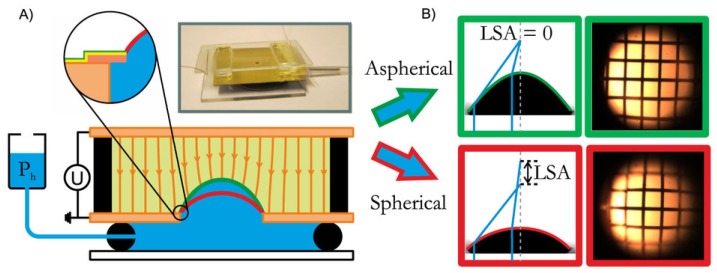
(**A**) Schematic of the aspherical lens device with a photograph of the actual device in the inset. Aqueous cesium iodide solution as lens fluid (in blue) with silicone oil as ambient liquid (in yellow). The curvature of the spherical meniscus (in red) at zero voltage is controlled by the hydrostatic head (in black). Application of voltage ‘U’ between the top electrode and the aperture plate (both in orange) distorts the shape of the spherical droplet, thus making a perfect aspherical lens. (**B**) The spherical (red) and aspherical droplet profiles (green) with captured images of the square grid demonstrate the mitigation of longitudinal spherical aberration (LSA). Reprinted by permission from Macmillan Publishers Ltd.: *Scientific Reports*, Mishra *et al.* [65], copyright 2013.

However, truly adaptive optics requires working with liquid lenses so that the focal length can be tuned back and forth by the application and release of driving pressure. Mishra *et al.* [65] therefore recently exploited insights from recent numerical simulations ([62]), to experimentally demonstrate an optofluidic lens with tunable focal length and asphericity. The researchers showed that two control parameters, the electric field and the hydrostatic pressure, can be used to tune the asphericity and focal length independently. The device is illustrated in Figure 8A. Simultaneously varying the hydrostatic pressure and voltage achieves a hyperbolic lens profile of the liquid–liquid interface with reduced longitudinal spherical aberration (LSA). Silicone oil is used as an insulating ambient fluid while aqueous salt solution is used in the droplet phase. The refractive index ratio was 1.10. Because of the density-matched system, the bond number is low, and thus, the effect of gravity can be neglected. Conducting experiments in fluidic ambience also arrests evaporation. Droplet interface profiles extracted experimentally are confirmed with electro-fluidic simulations using COMSOL Multiphysics. Further, they successfully demonstrated the concept by imaging a square grid. Not only is the LSA mitigated but also, in the process, distortion is suppressed, restoring the flat topography of the original grid (Figure 8B). The voltage required ranged from several hundred volts up to more than 1 kV. However, the voltage can be reduced by optimizing the device configuration with more rigorous numerical analysis. This includes choosing liquids with lower interfacial tension, enlarging the aperture size, and reducing the spacing between the aperture plate and the top electrode.

The same concept can also be extended to arbitrarily shaped electrodes that replace the flat plate electrode above the lens. Lima *et al.* [61] recently characterized the optical performance of an astigmatic lens by studying the variation in the corresponding Zernike coefficient on Zemax. The astigmatic lens is created by subjecting the liquid meniscus to an electric field using a stripe electrode. It was shown that the maximum tuning range of astigmatism is achieved when the stripe width of the electrode is half of the aperture diameter. Cylindrical liquid crystal lenses [66] are fabricated by applying a spatially non-uniform electric field on the homogenous liquid crystal. This is achieved by using a stripe ITO electrode as the top substrate with liquid crystal resting on the planar bottom substrate. Applying voltage deforms the lens along the stripe, thus creating a directional strain. Miccio *et al.* [67] exploited the concept of pyro-electrowetting for fabricating hemicylindrical and toroidal liquid lenses, followed by their interferometric characterization.

Electrically driven aspherical lenses come with a significant advantage over regular spherical lenses. Such lenses are not only tunable in their focal length, but can also be tuned independently for correcting optical aberrations. Thus, their inclusion can improve the overall performance of the optical system.

### 4.2. Thermally Driven Lenses

Lee *et al.* [68] fabricated PDMS microlenses with tunable astigmatism by exploiting the anisotropic joule heating produced by passing current through an elliptical silicon ring enclosed at the center of the PDMS lens. The applied current deforms the polymeric material asymmetrically. The lens can be tuned reversibly by turning the heat on and off. The astigmatic focal distance can be changed from 1590 to 44 μm by increasing the input current from 0 to 30 mA. Subjecting the lens polymeric material to heating and cooling cycles deteriorates the lens fabric.

### 4.3. Mechanically Driven Lenses

**Figure 9:**
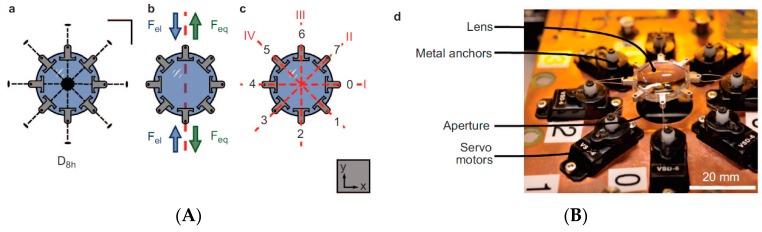
(**A**) (**a**) Schematic of elastomeric polymer anchored lens; (**b**) one of the actuation axes (in red dashed lines). F_eq_ denotes the applied radial force required to change the curvature of the lens while F_el_ represents the restoring force due to polymeric lens material. (**c**) Labeling of the four actuation axis. (**B**) Picture of the anchored lens with servo motors. Reprinted by permission from Macmillan Publishers Ltd.: *Nature LSA*, Liebetraut *et al.* [18], copyright 2013.

Liebetraut *et al.* [18] (Figure 9) demonstrated that applying the azimuthal asymmetric strain in a controlled manner on an elastomeric PDMS lens can reversibly switch the astigmatism on and off, simultaneously tuning the focal length. The lens can be astigmatically tuned by anisotropic actuation, by applying radial pressure along the four independent axis. This is a significant improvement over previously reported approaches [68], in which a deformable soft polymer is used to modulate the focal length under the applied stimuli. In these cases, the actuation was unidirectional and was possible only along one axis. However, the current approach of mechanical strain offers actuation possibilities along multiple directions. Figure 9 shows the schematic of the rigidly anchored PDMS lens attached with eight actuators, each controlled by a servo motor. The unstrained initial focal length of the PDMS lens is 32.6 mm. The lenses were optically characterized by an SHWS by studying the wavefront errors of first 36 Zernike coefficients. Astigmatism can be tuned over a range of 3 μm and focal length over +2.3 mm.

### 4.4. Hydrodynamically Driven Lenses

Yu *et al.* [69] designed a PDMS-encapsulated, adaptive liquid lens with one aspherical surface. The other surface, which is spherical, is modulated by fluidic pressure. Simulations conducted on Zemax show that the lens configuration significantly reduces the spherical aberration more than plano-convex lenses. The optimized spherical aberration was found to be −0.000059 waves compared to 2.03 waves for spherical lenses at optimized focus. The corresponding P-V and RMS of the wavefront error were also substantially reduced. Zhao *et al.* [70] devised a procedure for developing an endoscopic microscope by combining two liquid tunable aspherical lenses along with conventional customized plano-convex lenses. Tunability precludes any type of longitudinal translation between individual lens elements. The inter-element distance is optimized by the optical simulation platform Zemax. Moreover, the combined lens system acts as a potential zoom lens, offering a much larger field of view (FOV) and simultaneously ensuring high optical resolution. This fosters the imaging potential in endoscopic operations, as one needs to discern fine details with sufficient zoom while simultaneously capturing a large area for visualization. The performance of the composite optical system is quantified by measuring the MTF. For optimized aspherical lens parameters, the MTF of a system approaches the diffraction-limited MTF curve. However, pertinent literature and scientific investigations in devising such aspherical lens systems are still scarce. Mao *et al.* [71] designed and fabricated hydrodynamically tunable optofluidic cylindrical microlens by utilizing the interface between the laminar streams of 5 M calcium chloride and deionized water with a refractive index contrast. The interface experiences centrifugal force as the streams pass through the curved trajectory. Optimal calibration of the flow rates of the streams can be used to tune the focal length of the interfacial cylindrical lens. Higher flow rates result in shorter focal lengths. Zhao *et al.* [72] constructed a cylindrical microlens array by manually translating the piston, which, in turn, pushes the fluid, creating the liquid lens array in drilled apertures. This offers a higher dynamic range and is capable of characterizing a highly aberrated wavefront. Marks *et al.* [73] constructed an adaptive fluidic phoropter consisting of astigmatic and defocus lenses, designed for ophthalmic applications. The phoropter offers advantages over the conventional customized phoropter by decreasing the eye inspection time and being more compact and handy because of the considerably reduced size.

## 5. Outlook: Advanced Optofluidic Imaging Systems for the Future

The quality of optical images is frequently limited by the aberrations introduced either by the imaging optics or by the sample of interest, as for instance in microscopy applications. The primary benefit of micro-optical systems is their compactness. Unlike macroscopic optical systems, it is usually not possible to introduce additional optical elements to compensate aberrations without sacrificing this primary benefit of the approach. Adaptive micro-optics with aberration control offers a great opportunity to overcome this bottleneck and develop high-quality imaging optics for confined spaces with flexible aberration control. The recent examples sketched in the preceding sections should be considered as a first indication of the potential that these techniques can offer. Elastomeric lenses such as the one described in [18] already provide a great degree of flexibility and control over a variety of independent types of aberrations. Challenges for device development and true micro-optical integration of such devices include stability of the materials over (hundreds) of thousands of actuation cycles and the miniaturization of the external mechanical actuators. The electrically actuated lenses with aberration control seem to be more flexible in that respect. Miniaturization is not an issue because the actuation only requires patterned electrodes with dimensions of tens of micrometers that are easy to fabricate and connect. Long-term stability of the material is an issue, yet extensive developments in the context of commercially available EW-adaptive lenses (e.g., by Parrot/Varioptic Inc.) and display technology (e.g., Amazon/Liquavista) demonstrate the existence of reliable solutions to these problems. A technical concern, however, may arise from the rather high voltages of several hundred volts that had to be used in the first demonstrators [65]. Many applications will probably benefit optimized designs with reduced voltages.

Notwithstanding the practical challenge of high voltages, the degree of flexibility offered by electrically actuated lenses is tremendous. It is easy to extend the concepts of [65] to arbitrary electrode geometries that enable flexible control of a large spectrum of aberrations. The numerical study by Lima *et al.* [61] demonstrates this for a simple stripe electrode that introduces astigmatism. Yet, by making use of arrays of electrodes and even wider degree of flexibility can be achieved. Designs with, say, 10 × 10 or 100 × 100 individually addressable electrodes are easy to design and manufacture. Thanks to the quantitative numerical models that are available, systems can be designed to match the needs. Given the arbitrary number of actuators, such devices may eventually offer an even higher degree of flexibility than elastomeric devices. For instance, it is anticipated that the combination of pressure control and individually addressable segmented electrodes will enable the creation of lenses with surface profiles that vary between regions with positive and negative curvature within the same lens to generate almost arbitrary shapes of focal spots. In combination with suitable online wavefront metrology and self-learning algorithms, such devices could provide hitherto unimaginable flexibility of wavefront control.

One important challenge to introducing liquid lenses into a broader range of application fields would be the scaling up of their dimensions. For standard microscopy applications (including confocal), apertures of several (>5 mm) millimeters would be highly desirable. Control of gravitational and other distortions by suitable electric fields applied to segmented electrodes may offer a route to overcome such challenges in future devices.

**Figure 10:**
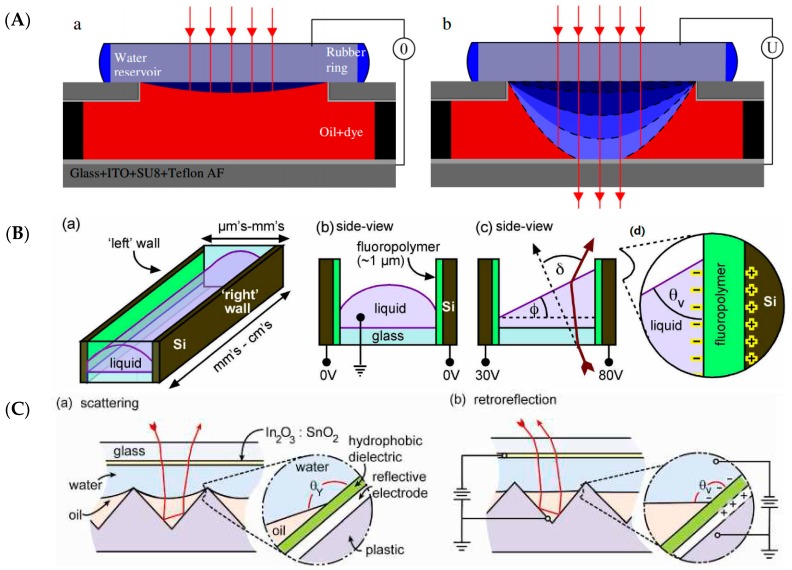
(**A**) (**a**) Incident light beam is absorbed by the “Oil + dye” medium; (**b**) application of voltage “U” exerts electrical Maxwell stress on the water-oil meniscus, enabling the incoming light to pass through the “Oil + dye” medium. With kind permission of the Optical Society of America (OSA). Adapted from Murade, C.U.; Oh, J.M.; van den Ende, D.; Mugele, F. Electrowetting driven optical switch and tunable aperture. *Opt. Express* **2011**, *19*, 15525–15531. (**B**) Schematic of the electrowetting modulated microprisms. (**a**) Conducting liquid entrapped between the silicon walls coated with fluoropolymer; (**b**) side view of the setup at zero voltage; (**c**) application of voltage, with 30 V (left sidewall) and 80 V (right sidewall), entrapped conducing liquid electrowets the sidewalls, thus forming a triangular prism; (**d**) inset of the contact line at the edge. With kind permission of the Optical Society of America (OSA). Adapted from Smith, N.R.; Abeysinghe, D.C.; Haus, J.W.; Heikenfeld, J. Agile wide-angle beam steering with electrowetting microprisms. *Opt. Express* **2006**, *14*, 6557–6563. (**C**) Schematic of the retroreflector. (**a**) Concave interface is formed between the low index water and high index oil at zero voltage, exhibiting scattering or semi-diffused reflection. Inset depicting the contact angle θ_Y_ between water, oil, and hydrophobic dielectric deposited on reflective electrode. (**b**) Transition from concave to flat interface at 19 V showing retroreflection. With kind permission of the Optical Society of America (OSA). Adapted from Kilaru, M.K.; Yang, J.; Heikenfeld, J. Advanced characterization of electrowetting retroreflectors. *Opt. Express* **2009**, *17*, 17563–17569.

The strength of such adaptive micro-lenses can be further enhanced by integrating them with other adaptive micro- and optofluidics devices for light manipulation that have been developed in recent years in parallel with the advances in optofluidics lenses discussed in this work. Figure 10 illustrates a few examples of such devices that can be integrated into various optical devices for a wide range of applications, from photonics [74], display technologies [75], and the biomedical industry to integral imaging for 3D vision [76]. These examples include elements such as shutters [77], beam steering prisms [78], and controllable reflectors [79]. All examples shown make use of the enormous flexibility of electrowetting to manipulate the shape and orientation of fluid interfaces.

Figure 10A shows a shutter of a variable circular aperture that can be tuned between 0.2 and 1.2 mm in diameter on a time scale of a 2 ms for the opening and of approximately 100 ms for switching off. This strong asymmetry is governed by the fact that the switching-off process in this device is driven by capillary forces only. *i.e.*, it is not supported by EW. Figure 10B shows an EW-actuated microprism with a flat (uncurved) liquid–liquid interface of variable tilt [78] that enables steering of beams in two independent directions on a time scale of ms. Similarly, wedge-shaped geometric structures can be exploited to create efficient switchable retroreflectors [79] by alternating between a flat and a curved liquid–liquid interface, Figure 10C. Recently, Schuhladen *et al.* [80] constructed a tunable optofluidic slit aperture actuated by AC electrowetting.

Other future developments may arise in the area of integral imaging for 3D vision. While tunable optofluidic microlens arrays have been demonstrated, their application to enhance the accessible focal depth of 3D imaging systems has yet to be explored. If combined with aberration control, as discussed above, such devices might lead to breakthroughs in quantitative 3D metrology, e.g., in industrial applications such as quality control.

## 6. Conclusions

The review summarized the classification of optofluidic lenses based on their shapes and driving mechanisms and briefly sketched the standard tools employed for their characterization. The main focus is on two aspects of lens performance: tunability of the focal length and the more recent developments on aberration control. Liquid spherical lenses, although tunable in the focal length, are inherently compromised by optical aberrations. Thus, these lenses do not improve the optical quality of the captured image. Further, solid aspherical lenses are expensive and require high mechanical precision in their fabrication. Moreover, they are designed for a single focal point. Tunable aspherical lenses, with a reversibly tunable focal length, are promising candidates for suppressing spherical aberration and for improving optical resolution. Liquid lenses can also be a substitute for GRIN lenses. By choosing suitable fluid compositions and controlling their dispersion, one can minimize chromatic aberrations. We also discussed various lens tuning mechanisms. Each mechanism has a distinct operational procedure and has its own set of advantages and disadvantages; for example, actuation mechanisms involving microfluidic vents for directing liquids have a long response time. This may be due to the high fluidic resistance encountered in microfluidic systems. Similarly, for thermally actuated lenses, there is a considerable delay between the heating and cooling cycles, due to the low coefficient of thermal expansion, and thus, there is a lag in response. However, these approaches offer better control over lens morphology by carefully regulating the temperature, for thermally actuated lenses, or the fluid pressure, for hydrodynamically manipulated lenses. Electric fields offer a more detailed and faster control. Companies such as Liquivista and Varioptic/Parrot manufacture reliable electrowetting devices that have a long shelf life, demonstrate excellent performance over continuous operation, and last for multiple actuation cycles. The application of an electric field also provides an inexpensive approach for designing tunable liquid aspherical lenses with a minimum of mechanical actuators. Electrically actuated liquid lenses enable the scanning of objects at varying distances and simultaneously compensate for spherical aberrations. Despite the technological flexibility that has been demonstrated for a few examples, aberration control still requires rather high voltages, typically several hundreds of volts. Overall, the development of devices with aberration control—be it with electrical actuation or with elastomeric lenses—is still in its infancy and deserves more attention and optimization. Improving robustness and fabrication procedures as well as the long-term stability of the devices are additional aspects that need to be addressed.

## Figures and Tables

**Table 1:** Classification of Zernike polynomials and corresponding optical aberration. Even and odd Zernike polynomials are defined as $Z_{n}^{m}=R_{n}^{m}\left(\rho \right)\cos\left(m\phi \right)$ and $Z_{n}^{m}=R_{n}^{m}\left(\rho \right)\sin\left(m\phi \right)$ , where ρ and ϕ are the radial and azimuthal coordinate and $R_{n}^{m}\left(\rho \right)=\sum {\left(-1\right)}^{k}\left(n-k\right)k!\left[k!\left(\frac{n\;+\;m}{2}-k\right)!\left(\frac{n\;-\;m}{2}-k\right)!\right]\;\rho^{n\;-\;2k}$ is the radial function for (n−m)≥0 and even. The sum runs from 0 to (n−m)/2 . For (n−m) odd: $R_{n}^{m}\equiv 0$.

Index	Radial Nodes (n)	Azimuthal Index (m)	Type of Aberration
1	0	0	piston
2	1	1	X-tilt (tip)
3	1	−1	Y-tilt (tilt)
4	2	0	Defocus
5	2	−2	Oblique astigmatism
6	2	2	Vertical astigmatism
7	3	−1	Vertical coma
8	3	1	Horizontal coma
9	3	−3	Vertical trefoil
10	3	3	Oblique trefoil
11	4	0	Primary spherical
12	4	2	Vertical secondary astigmatism
13	4	−2	Horizontal secondary astigmatism
14	4	4	Vertical quadrafoil
15	4	−4	Oblique quadrafoil
